# Repeated sprint training induces prolonged residual fatigue compared to other high-intensity interval training modalities in middle-distance runners

**DOI:** 10.5114/biolsport.2026.156227

**Published:** 2025-11-24

**Authors:** Kai Yang, Yang Xia, Ana Silva Filipa

**Affiliations:** 1 School of Sports Science Research, Nanjing Normal University, 210041, Nanjing, China; 2 Department of Physical Education, High School Affiliated to Nanjing Normal University, 210000, Nanjing, China; 3 Sport Physical Activity and Health Research & Innovation Center, Rio Maior, Portugal

**Keywords:** Athletics, Interval training, Recovery, Fatigue, Training

## Abstract

This study aimed to monitor the kinetics of neuromuscular fatigue and inflammation indices in middle-distance runners following exposure to three different high-intensity interval training (HIIT) modalities: short-interval HIIT, long-interval HIIT, and repeated sprint training (RST). A crossover repeated-measures design was used involving 33 male middle-distance runners (19.6 ± 2.3 years) who completed short-interval HIIT, long-interval HIIT, and RST. Neuromuscular performance was assessed using the countermovement jump (CMJ) and isometric mid-thigh pull (IMTP), while inflammatory (salivary IL-6) and perceptual markers (delayed onset muscle soreness, DOMS and perceived recovery scale, PRS) were measured at rest, immediately post-exercise, and at 24 and 48 hours post-exercise to evaluate changes over time. All variables showed significant main effects of time and modality, as well as interactions (p < 0.001). Immediately post-exercise, RST consistently induced the greatest acute fatigue, evidenced by larger declines in CMJ (9.6%) and IMTP (6.7%), an increase in IL-6, and a 56.8% drop in PRS. At 24 hours post-exercise, RST still showed significantly reduced performance (CMJ and IMTP) and elevated inflammation (IL-6) compared to short and long-HIIT. DOMS peaked at 24 hours in the RST group, which also reported lower perceived recovery. By 48 hours, performance and inflammatory markers largely returned to baseline across all groups. However, RST continued to show higher DOMS and lower PRS than both short and long-HIIT (p < 0.001). These findings suggest that RST induces a more pronounced and prolonged recovery period compared to both short- and long-interval HIIT. Coaches should consider that recovery following RST sessions may require up to at least 48 hours, which is longer than for short- or long-interval HIIT.

## INTRODUCTION

High-intensity interval training (HIIT), in both submaximal and maximal modalities, has become a popular approach in sports that demand improvements in cardiorespiratory fitness, anaerobic capacity, and power development [1]. Due to the intense effort involved and the time-efficient nature of these workouts, HIIT modalities are particularly appealing for middle-distance runners (e.g., 800 to 5000 meters), who must sustain high speeds over moderate distances [2]. In these events, both metabolic and neuromuscular development are critical to enhancing performance and increasing the likelihood of success [3]. Specific HIIT protocols—such as short- or long-interval HIIT targeting maximal oxygen uptake (${\dot{\text{V}}\text{O}}_{2\max}$), or repeated sprint training (RST) focused on anaerobic performance—can provide a sport-specific stimuli that align with the physiological demands of middle-distance running [2].

However, the volume and modality of HIIT can influence its impact on athletes. For example, a typical short-interval HIIT protocol may involve 6–8 repetitions of 300 m runs at 115–120% of maximal aerobic speed (${\text{v}\dot{\text{V}}\text{O}}_{2\max}$), interspersed with 3–5 min of passive or light active recovery [4]. A long-interval HIIT session may consist of 12–15 repetitions of 300 m runs at ~95% of ${\text{v}\dot{\text{V}}\text{O}}_{2\max}$, or 4 repetitions of 1500 m at ~90–95% of ${\text{v}\dot{\text{V}}\text{O}}_{2\max}$, each separated by 2–4 min of recovery [4]. In contrast, repeated sprint training (RST) emphasizes anaerobic and neuromuscular stress, with 2–4 sets of 4–6 maximal sprints of ~20 m (≤ 5 s each), separated by 20–30 s of recovery between sprints and 3–4 min between sets [5]. RST not only places high metabolic demands on the athlete but also delivers a strong neuromuscular stimulus [5]. Given the high intensity of these training formats, the resulting acute physical stress may lead to residual fatigue, potentially affecting recovery in the short to medium term following the session.

The impact of HIIT on the neuromuscular response of middle-distance runners has been explored. In one study [6], athletes performed five 300-meter repetitions at intensities corresponding to approximately 77% of their maximal sprint speed and 120–130% of their velocity at ${\dot{\text{V}}\text{O}}_{2\max}$ (${\text{v}\dot{\text{V}}\text{O}}_{2\max}$), with 1-minute active recovery periods consisting of a 100-meter walk-jog. While this protocol did not significantly alter maximal voluntary contraction torque of the knee extensors, it did result in a decline in excitation–contraction coupling efficiency, indicating a neuromuscular impact despite unchanged maximal force output [6]. In contrast, another investigation involving long-interval HIIT—comparing 1-minute work/1-minute rest and 2-minute work/2-minute rest formats at ${\text{v}\dot{\text{V}}\text{O}}_{2\max}$—found no significant changes in countermovement jump performance across conditions, suggesting minimal acute neuromuscular fatigue under these contexts [7]. Regarding RST, evidence indicates that it can lead to pronounced muscle fatigue, particularly when a high number of sprint repetitions are completed, as reflected by reduced repeated-sprint performance [8].

While the acute neuromuscular effects of different HIIT modalities are relatively well documented, the residual fatigue and recovery timeline following these sessions are less thoroughly explored—particularly in middle-distance runners. For instance, Astorino et al. observed delayed heart rate and ${\dot{\text{V}}\text{O}}_{2}$ recovery during cycling-based HIIT protocols after bouts of 30–60 seconds [9]. Chorley et al. [10] also documented that power output in trained cyclists during severe HIIT intervals was modulated by recovery intensity, indicating that the recovery period between high-intensity efforts is an important determinant of residual performance. Understanding the time course of recovery is critical for planning training. Some preliminary findings suggest that HIIT can immediately disrupt neuromuscular control, leading to altered gait kinematics, increased coordination variability, and impaired muscle function shortly after exercise [11]. However, another study [12] has reported minimal changes in running mechanics despite high levels of exertion, suggesting that the type of HIIT protocol, the athlete’s conditioning level, and task specificity may contribute to the variability in neuromuscular responses. Moreover, research involving athletes from other sports indicates that neuromuscular recovery can extend up to 72 hours post-HIIT, as evidenced by delayed normalization of performance markers such as countermovement jump height and reactive strength index [13]. This variability highlights the need for more investigation into how different HIIT formats affect fatigue and recovery in runners, using both performance-based and physiological markers.

Neuromuscular performance in distance runners depends not only on the ability to generate maximal force during brief ground contact times but also on maintaining consistent voluntary muscle activation throughout repeated efforts. High-intensity interval training (HIIT), depending on its structure and intensity, can impair these neuromuscular functions by inducing both central and peripheral fatigue. Certain HIIT modalities may also trigger an inflammatory response, particularly when mechanical and metabolic stress is high. In this context, combining neuromuscular indicators (e.g., countermovement jump [CMJ], isometric mid-thigh pull [IMTP]) with inflammatory biomarkers such as salivary interleukin-6 (IL-6) provides a more comprehensive understanding of the physiological demands imposed by different HIIT formats.

Residual fatigue refers to the persistence—beyond the immediate post-exercise period—of both performance fatigability (objective reductions in maximal force/power) and perceived fatigability (soreness/low recovery), typically lasting hours to days after training [14]. To capture this, we assessed performance fatigability with countermovement jump (CMJ) and isometric mid-thigh pull (IMTP), which are reliable and sensitive indices of transient neuromuscular impairment [15, 16]. Salivary IL-6 was included as a biomarker of physiological load because it is released by skeletal muscle in response to intense contractile and metabolic stress, acts as a key myokine mediating energy mobilization and inflammatory signaling, and has been shown to remain elevated during the recovery period following high-intensity exercise [17]. Thus, IL-6 offers a non-invasive marker for monitoring the inflammatory component of residual fatigue. Finally, we quantified perceived fatigability with delayed onset muscle soreness (DOMS) and the Perceived Recovery Status (PRS) scale— tools shown to track recovery status over 24–72 h [18]. In particular, perceptual tools such as DOMS and the PRS scale may offer coaches and athletes an accessible, non-invasive means to monitor recovery status in daily training environments, complementing laboratory-based assessments. These measures provide immediate feedback on how athletes feel and perceive their readiness, thereby supporting individualized decisions regarding training load adjustment and session planning.

Although several studies have examined inflammatory or muscle damage biomarkers in response to HIIT [19, 20], and others have attended to performance or neuromuscular outcomes, few have simultaneously tracked both neuromuscular (e.g., CMJ, IMTP) and inflammatory markers across different HIIT modalities within the same cohort. Systematic reviews [21] confirm this gap. Addressing this combination, namely multiple modalities, multidimensional outcome measures and middle-distance running population will help clarify how modality influences residual fatigue and recovery kinetics. By incorporating both objective and subjective measures at delayed time points (e.g., 24 and 48 hours post-session), it becomes possible to evaluate the cumulative impact of various HIIT modalities on recovery kinetics. This integrated approach helps identify how different protocols influence readiness in middle-distance runners, thereby informing training prescription and periodization strategies. Therefore, this study aimed to determine the neuromuscular fatigue and inflammation in middle-distance runners following three distinct HIIT modalities: short-interval HIIT, long-interval HIIT, and repeated sprint training (RST). Assessments were performed at rest, immediately after the training session, and at 24 and 48 hours post-exercise using CMJ, IMTP, salivary IL-6, DOMS, and PRS.

## MATERIALS AND METHODS

### Study design and experimental approach

This study employed a crossover repeated-measures design in which all middle-distance runners participated in three different HIIT modalities: short-intervals, long-intervals, and RST. The order of participation in each modality was randomized to minimize sequencerelated bias in the measured parameters. The study was conducted over three consecutive weeks. During this period, each athlete completed one HIIT session per week, scheduled on Mondays following a 48-hour rest period over the weekend. This timing allowed athletes to begin each week in a rested state. No additional washout period beyond this 48-hour rest was imposed, as participants continued their regular training schedules between testing weeks. This design was selected to balance ecological validity with athlete availability. After each Monday HIIT session, athletes were given another 48 hours of rest before resuming their regular training routines. Assessments were conducted at four time points: at rest (prior to the HIIT session), immediately after the session, 24 hours post-session, and 48 hours post-session. Data collection took place during April and May 2025 and was carried out in small groups over an extended period to maximize participation and ensure appropriate data acquisition. As soon as a volunteer was recruited, they were scheduled for monitoring across the three-week protocol. The HIIT sessions were designed and scheduled by the research team, while evaluators remained blinded to the specific HIIT modality each athlete was assigned at any given time.

### Participants

To conduct the study, an a priori inclusion criteria was established, namely identifying participants who fulfilled the following aspects: (i) being a middle-distance runner with at least 3 years of experience; (ii) being male (only male runners were recruited to reduce variability associated with sex-specific hormonal fluctuations and neuromuscular responses, thereby improving sample homogeneity); (iii) being older than 16 years; (iv) not being injured in the month prior to the study or during the experimental period; (v) not being in a competition phase throughout the experiment; (vi) refraining from training on weekends and for 48 hours after each HIIT session; (vii) participating in all scheduled assessment time points and HIIT sessions. As exclusion criteria, participants were classified as ineligible if: (i) they consumed any type of drugs or illicit supplements; (ii) their regular training schedule overlapped with the designated rest periods of the experiment. Recruitment was conducted by approaching local athletics clubs and inviting technical directors and athletes to participate.

An a priori estimation of sample size was conducted for a twoway repeated measures ANOVA, in which the same participants took part in three conditions, with measurements taken at four time points for each condition. Assuming an effect size (f) of 0.2 and a statistical power of 0.85, the a priori estimation for repeated measures within factors recommended a total sample size of 29 participants. The sample size calculation was performed using G*Power software (version 3.1). A previous study [22], which also compared different HIIT training regimens and their immediate neuromuscular impact, tested a sample of 31 participants, thereby closely aligning with this number.

After an initial recruitment of 37 middle-distance athletes, 4 were excluded because they could not ensure the required 48-hour rest period following the HIIT sessions. Based on this preliminary information provided by the athletes, they were not included in the experimental phase. Therefore, a total of 33 middle-distance runners were included in the sample, all of whom completed evaluations at every time point. Participants were randomly allocated to one of the following intervention sequences: (i) short-HIIT (week 1) – long-HIIT (week 2) – RST (week 3); (ii) long-HIIT (week 1) – short-HIIT (week 2) – RST (week 3); (iii) short-HIIT (week 1) – RST (week 2) – long-HIIT (week 3); (iv) long-HIIT (week 1) – RST (week 2) – short-HIIT (week 3); (v) RST (week 1) – short-HIIT (week 2) – long-HIIT (week 3); (vi) RST (week 1) – long-HIIT (week 2) – short-HIIT (week 3). Randomization was conducted using a simple random procedure with a balanced number of opaque envelopes which hold the specific number of sequences that would be made. Athletes selected an envelope at the time of their initial demographic and anthropometric assessment. Participants were, on average, 19.6 ± 2.3 years old, with a body mass of 63.7 ± 3.6 kg, a height of 174.1 ± 4.0 cm, and a body mass index of 21.0 ± 0.7 kg/m^2^. Their ${\dot{\text{V}}\text{O}}_{2\max}$ during the five-minute track running test was 5.00 ± 0.16 m/s, covering an average distance of 1498.8 ± 47.5 meters. They typically engaged in five to six training sessions per week during their regular practice routines. However, during the study period, they adjusted their training schedules to comply with the required rest periods following HIIT sessions.

The study was conducted by directly approaching the athletes. In the case of participants under 18 years of age, their parents were also approached. After being informed about the study, participants (and their parents, when applicable) signed a free and informed consent form to ensure compliance with all ethical standards. Additionally, the study received ethical approval from the Nanjing Normal University Biomedical Ethics Committee (approval code NNU202410003)

### High-intensity interval training interventions

The HIIT sessions were conducted at the same facility—the track where the athletes regularly trained. These sessions took place between 3:30 and 5:30 p.m., depending on athlete availability. However, to ensure consistency, each athlete always trained at the same time across all conditions. Every HIIT session was preceded by a standardized warm-up for all participants, which included 10 minutes of light jogging, followed by dynamic stretching and mobility drills targeting the hips, hamstrings, calves, and quadriceps (e.g., leg swings, walking lunges, hip circles, and arm rotations). After mobility, athletes performed running drills such as high knees, butt kicks, and straight-leg bounds. The warm-up concluded with three progressive accelerations over 30 meters. A researcher supervised the entire warm-up and also provided instructions for the HIIT session. Following the warm-up, athletes rested for three minutes before beginning the workout. The HIIT sessions were conducted continuously on the track — without turning points or changes of direction. Audio signals provided by the researcher guided the athletes’ pace, ensuring adherence to the required intensity for both short and long-HIIT formats. Specific points were marked on the track to help athletes maintain proper pacing and synchronize their efforts with the audio cues.

The short-HIIT protocol consisted of 12 repetitions of 200-meter runs at 120% of ${\text{v}\dot{\text{V}}\text{O}}_{2\max}$, with 3-minute recovery intervals between each repetition. The long-HIIT protocol involved 4 repetitions of 1000-meter runs at 95% of ${\text{v}\dot{\text{V}}\text{O}}_{2\max}$, also with 3-minute recovery intervals between runs. The RST protocol included all-out 20-meter sprints, each followed by 20 seconds of jogging. Each set consisted of 6 sprints, with a total of 4 sets performed. Recovery between sets was 5 minutes. The ${\text{v}\dot{\text{V}}\text{O}}_{2\max}$ was estimated using a 5-minute track running test, which has been previously validated and shown to be a reliable method for estimating ${\text{v}\dot{\text{V}}\text{O}}_{2\max}$ when compared to laboratory treadmill tests using a gas analyzer [23]. This test has also showed good reliability [24], confirming its accuracy in determining training intensity for athletes. The 5-minute test was conducted during the week prior to the start of the study, as part of a specific session in which demographic and anthropometric data were also collected. During the test, athletes were instructed to run at their maximal sustainable pace for 5 minutes on a standard track. To assist athletes, they received audio feedback every minute and again during the final 30 seconds, ensuring they were aware of the remaining time. The total distance covered during the 5 minutes was recorded and divided by the total number of seconds to calculate ${\text{v}\dot{\text{V}}\text{O}}_{2\max}$, which was then used to prescribe both short- and long-HIIT training intensities.

The ${\text{v}\dot{\text{V}}\text{O}}_{2\max}$ was assessed once at baseline using the field test, and this value was used to prescribe intensities for all HIIT sessions. To reduce variability, all training sessions were performed under comparable environmental conditions and at the same time of day. Athletes were instructed to maintain their normal training routines, and no re-testing of ${\text{v}\dot{\text{V}}\text{O}}_{2\max}$ was conducted between modalities.

### Procedures for Measurement

Measurements were conducted at four different time points, always following the same sequence of tests and procedures to minimize the influence of changing conditions. The first set of measurements was taken at rest, before the start of the HIIT session. The HIIT session began five minutes after the last test in this initial assessment. Five minutes after completing the HIIT session, the athletes underwent a second evaluation, following the same sequence of tests. Further assessments were conducted 24 hours and 48 hours after the HIIT session. To avoid interference from additional physical exercise (training), athletes were instructed to refrain from training during the recovery period. Each assessment began with the completion of the DOMS and PRS questionnaires, followed by the collection of saliva samples for interleukin-6 analysis. Athletes then performed a short warm-up consisting of three minutes of jogging and dynamic stretching of the lower limbs for the pre-exercise, 24 h, and 48 h assessments. At the immediate post-HIIT time point, no additional warmup was administered, as athletes were already in an elevated physiological state. After the warm-up (when applicable), they were assessed with three trials of the IMTP test, followed by three trials of CMJs. All evaluations were carried out in a private room near the track facilities, under similar environmental conditions (~22°C). A rest period of three minutes was provided between the IMTP and CMJ tests.

#### Delayed onset muscle soreness

Delayed onset muscle soreness (DOMS) in the lower limbs was assessed subjectively using a previously validated questionnaire [25]. The questionnaire consists of a Likert scale ranging from 0 to 6, where 0 indicates no muscle soreness and 6 represents severe pain that may limit movement. To ensure familiarity with the scale, participants were introduced to it during the week prior to the start of the study, specifically on the day of anthropometric assessments and the 5-minute performance test. The scale was translated and adapted to the local language, and its verbal anchors were refined based on expert validation to ensure accurate representation of the intended meanings. Participants completed the scale individually using a printed version designed to guide their responses. All scores were collected and recorded by a researcher to ensure consistency and accuracy in the assessment process.

#### Perceived recovery status scale (PRS)

The PRS scale was also introduced to the athletes, following the same familiarization procedures used for the DOMS scale. The PRS is a validated Likert-type scale in which a score of 0 indicates very poor recovery or extreme fatigue, 5 represents adequate recovery, and 10 (the maximum score) reflects very good recovery or high energy levels [26]. This scale has been previously validated and has shown sensitivity in detecting changes following repeated sprint exercises [26]. At each assessment time point, the PRS scale was presented to the athletes using a printed form that included clearly defined verbal anchors. These anchors were adapted to the local language and validated by subject-matter experts to ensure conceptual accuracy. Athletes provided their scores individually, and a researcher recorded the responses.

#### Salivary interleukin-6

The quantification of IL-6 (expressed in pg/mL) was performed using the ELISA kit specific for IL-6 (Salimetrics, USA), following the manufacturer’s protocol. Samples were analyzed in duplicate, aiming to mitigate for inter-assay variation. Unstimulated saliva samples were obtained from participants using a collection device (Salimetrics, USA), with each individual providing approximately 2 mL of fluid.

#### Isometric mid-thigh pull test

The IMTP was implemented to assess the maximal strength of the athletes. Each participant stood on a solid platform with a fixed bar positioned at mid-thigh height, connected to a crane scale. The testing position was individually adjusted to achieve approximately 145° of knee flexion and 145° of hip flexion, following previous recommendations to optimize force production [27]. Participants adopted an upright posture with a flat back, feet hip-width apart, and hands gripping the bar at shoulder-width using lifting straps to eliminate grip limitations. From this static position, the athlete pulled vertically as forcefully and rapidly as possible against the immovable bar for 5 seconds, with no actual movement occurring. All participants underwent a familiarization session with the IMTP procedures during the week prior to the study. They practiced the correct posture, bar grip, and pulling action under the researcher supervision to ensure proper technique and reduce potential learning effects.

Force output was measured using a RoMech crane scale with 0.05 kg increments, a precision of ± 0.1%, and a maximum capacity of 300 kg. The procedure followed a previous validation study, which confirmed that using a crane scale to measure IMTP is a valid method [28]. Each trial was recorded in slow motion using a mobile phone camera (iPhone 12, Apple Inc., USA; 240 fps, 1080p resolution) focused on the crane scale display, and the video files were analyzed using Kinovea motion analysis software (version 0.9.5, Kinovea.org) to extract the peak force values produced during the 5-second effort. Three trials were conducted, each separated by 30 seconds of rest. For each trial, the peak force value (in kilograms) was multiplied by 9.80665 to convert it to Newtons. This value was then divided by the athlete’s body mass (in kg) to obtain the standardized force output in N/kg. The average value across the three trials was recorded for each time point.

#### Countermovement jump test

A short repeated CMJ test was conducted for each participant, completing four CMJs, with a recovery of 3 seconds between each trial, aligning with a previous study employed to identify neuromuscular fatigue by means of CMJ [29]. During the jumps, players were instructed to keep their hands firmly on their hips, maintain an upright torso, avoid any forward leg movement during the flight phase, and try to land in the same spot they took off from, with their forefoot contacting the ground first. Participants were familiarized with the CMJ protocol in the week prior to the study, performing multiple practice jumps with standardized instructions (hands on hips, upright torso, forefoot landing) to ensure consistent execution across testing sessions.

Athletes were encouraged to use a preferred squat depth that felt most natural and effective for them, aiming to perform each jump as quickly as possible. This approach aligns with prior research showing that jump speed is the most critical factor for increasing CMJ height, while allowing a self-selected depth is sufficient to optimize performance [30]. CMJ height was recorded using the Optojump photoelectric measurement system (Microgate, Bolzano, Italy), which was connected to a laptop running Optojump software (version 1.10.19). This system captures flight time at a resolution of 1 millisecond (1 kHz). Using these flight times, jump height was calculated with the standard formula: (9.81 × flight time^2^) ÷ 8, as reported in the validation study of the platform [31]. The average height of CMJ height (cm) was recorded at each time point.

#### Secondary outcome – Rate of perceived exertion (RPE)

To monitor the intensity of the HIIT sessions, the Rate of Perceived Exertion (RPE) was assessed using the CR10 Borg scale approximately 20 minutes after each session. This scale, which has been previously validated for monitoring exercise intensity [32], is a Likerttype scale where 0 represents rest, 5 indicates hard effort, and 10 corresponds to maximal effort. Participants were already familiar with the scale, which was adapted to their language. As with other questionnaires, a researcher administered the scale and recorded the athletes’ responses to the question “On a scale from 0 to 10, where 0 means no effort at all and 10 means maximal effort, how hard did you feel you were working during the session?”. Participants were allowed to use intermediate values such as 0.5 or 2.5 to provide more precise ratings when they were uncertain between whole numbers.

### Statistical procedures

To examine the effects of different training modalities and time points on the measured outcomes, a two-way repeated measures ANOVA was conducted. This analysis considered two within-subject factors: Condition (Short-HIIT, Long-HIIT, and RST) and Time (Rest, Postexercise [Immediate], 24 hours post-exercise, and 48 hours postexercise). Prior to running the ANOVA, assumptions of normality, sphericity, and absence of significant outliers were tested. Normality was evaluated using the Shapiro-Wilk test (p > 0.05). Mauchly’s test of sphericity was used to assess the sphericity assumption; where sphericity was violated, the Greenhouse-Geisser correction was applied to adjust the degrees of freedom. Effect sizes were reported using partial eta squared (η^2^_p_), interpreted as small (≥ 0.01), medium (≥ 0.06), and large (≥ 0.14) [33]. Where significant main effects or interactions were found, post hoc pairwise comparisons were performed with Bonferroni correction to control for type I error. For the case of DOMS and PRS, since they are measured on an ordinal scale, non-parametric tests were performed, specifically the Friedman test, followed by multiple Mann-Whitney tests with Bonferroni correction to account for the number of comparisons made. All statistical analyses were conducted using IBM SPSS Statistics for Windows, Version 29.0 (IBM Corp., Armonk, NY, USA). The significance level was set at p < 0.05 for all tests.

## RESULTS

**FIG. 1:**
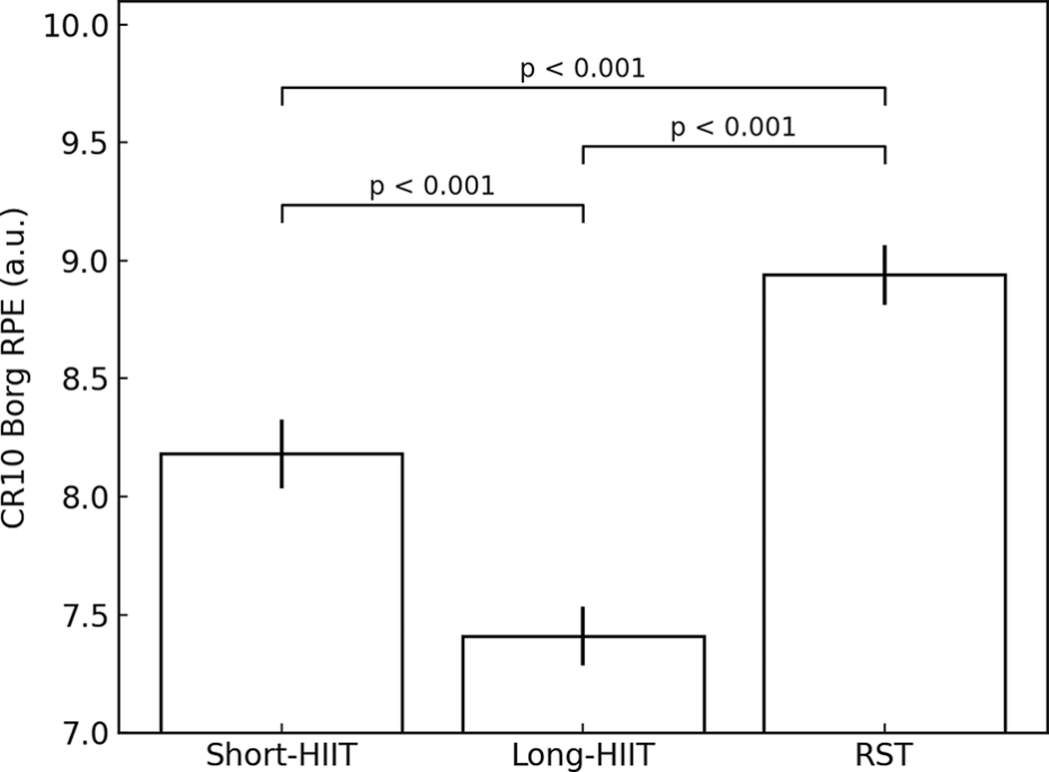
Mean rate of perceived exertion (RPE) scores and their 95% confidence intervals reported by athletes during short and long HIIT (high-intensity interval training) and repeated sprint training (RST) conditions.

Descriptive statistics for RPE across the three HIIT conditions are presented in Figure 1. Mean RPE was highest for RST (8.94 ± 0.37), followed by short-HIIT (8.18 ± 0.43), and lowest for long-HIIT (7.42 ± 0.38). A Friedman test was conducted to compare RPE across the three modalities, revealing a statistically significant difference, χ2(2) = 28.00, p < 0.001. Post hoc Wilcoxon signed-rank tests with a Bonferroni correction were performed for pairwise comparisons. The adjusted significance level for these comparisons was set at α = 0.05/3 = 0.017. Statistically significant differences were observed between the long-HIIT and short-HIIT protocols (Z = -4.816, p < 0.001 after Bonferroni correction). The rate of perceived exertion (RPE) was significantly higher in the RST protocol compared to both short-HIIT (Z = -4.789, p < 0.001 after Bonferroni correction) and long-HIIT (Z = -5.096, p < 0.001 after Bonferroni correction).

Table 1 presents the mean values ± standard deviations for each outcome variable across the three HIIT modalities (Short-HIIT, Long-HIIT, and RST) at different time points (Rest, Post-exercise, Post 24 h, and Post 48 h).

**TABLE 1:** Mean values ± standard deviations for each outcome variable across the three HIIT modalities (Short HIIT, Long HIIT, and RST) at different time points (Rest, Post-exercise, Post 24 h, and Post 48 h).

	IMTP (N/m)	CMJ (cm)	IL-6 (pg/mL)	DOMS (AU)	PRS (AU)
**Short HIIT**					
Rest	28.17 ± 2.54	40.97 ± 4.70	2.12 ± 0.25	0.00 ± 0.00	9.42 ± 0.50
Post-exercise	26.83 ± 2.45	37.60 ± 4.32	17.02 ± 1.12	0.21 ± 0.42	4.85 ± 0.91
Post 24 h	27.75 ± 2.48	39.45 ± 4.40	7.57 ± 0.82	3.42 ± 0.50	7.09 ± 0.91
Post 48 h	28.27 ± 2.59	40.62 ± 4.71	2.88 ± 0.41	1.94 ± 0.24	8.61 ± 0.66

**Long HIIT**					
Rest	28.13 ± 2.45	40.66 ± 4.45	2.22 ± 0.25	0.00 ± 0.00	9.33 ± 0.48
Post-exercise	27.39 ± 2.38	38.60 ± 4.23	18.18 ± 1.17	0.03 ± 0.17	5.70 ± 0.73
Post 24 h	28.10 ± 2.39	40.14 ± 4.34	7.25 ± 0.82	3.36 ± 0.78	7.61 ± 0.66
Post 48 h	28.09 ± 2.44	40.53 ± 4.41	2.46 ± 0.45	1.79 ± 0.54	8.61 ± 0.66

**RST**					
Rest	28.26 ± 2.50	40.89 ± 4.54	2.42 ± 0.25	0.00 ± 0.00	9.39 ± 0.50
Post-exercise	26.36 ± 2.45	36.95 ± 4.31	20.72 ± 1.22	0.21 ± 0.42	4.06 ± 0.79
Post 24 h	27.45 ± 2.46	39.08 ± 4.34	9.26 ± 0.81	4.03 ± 0.53	6.15 ± 0.87
Post 48 h	28.07 ± 2.53	40.46 ± 4.52	3.02 ± 0.41	2.42 ± 0.50	7.79 ± 0.78

HIIT: high-intensity interval training; RST: repeated sprint training; IMTP: isometric mid-thigh pull test; CMJ: countermovement jump test; IL-6: Salivary interleukin-6; DOMS: delayed onset muscle soreness; PRS: Perceived recovery status scale

### Isometric mid-thigh pull test

There was a significant main effect of time point on IMTP performance, F_(1.607, 51.411)_ = 70.39, p < 0.001, η^2^_p_ = 0.944. A significant main effect of HIIT modality was also found, F_(2, 64)_ = 16.45, p < 0.001, η^2^_p_ = 0.340. Additionally, a significant interaction effect between HIIT modality and time point was observed, F_(2.483, 79.446)_ = 33.02, p < 0.001, η^2^_p_ = 0.508.

**FIG. 2:**
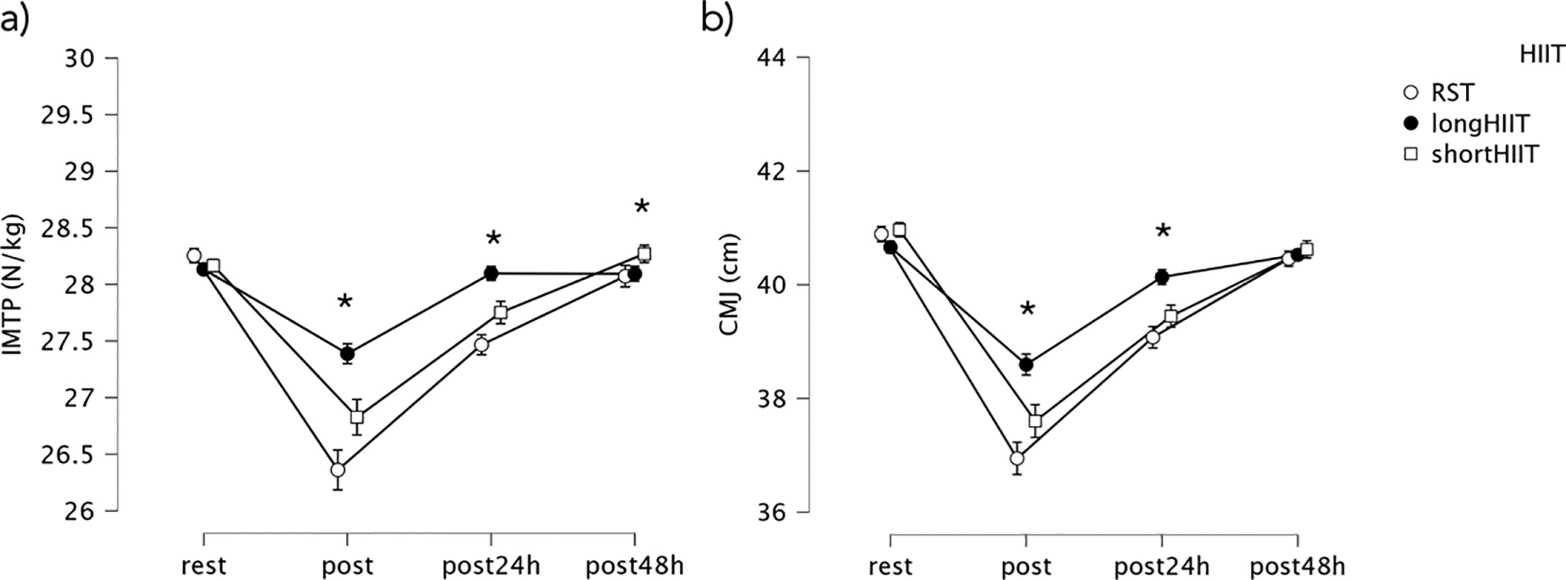
Mean values and 95% confidence intervals for variations in (a) isometric mid-thigh pull (IMTP) and (b) countermovement jump (CMJ) across the time points. * Indicates significant differences between groups at the corresponding time point (p < 0.05).

No significant differences were found between HIIT modalities at rest (p = 0.153). At the post-exercise time point, long-HIIT showed significantly lower IMTP compared to short-HIIT (mean difference = -0.56, p < 0.001). Long-HIIT also showed significantly lower IMTP compared to RST (mean difference = -1.03, p < 0.001), and RST showed significantly lower IMTP than short-HIIT (mean difference = -0.47, p = 0.001). At 24 hours post-exercise, short-HIIT continued to demonstrate significantly greater IMTP than both long-HIIT (mean difference = -0.35, p = 0.001) and RST (mean difference = -0.65, p < 0.001). At 48 hours post-exercise, short-HIIT still showed higher IMTP compared to RST (mean difference = -0.20, p = 0.026). Variations in IMTP are illustrated in Figure 2.

**FIG. 3:**
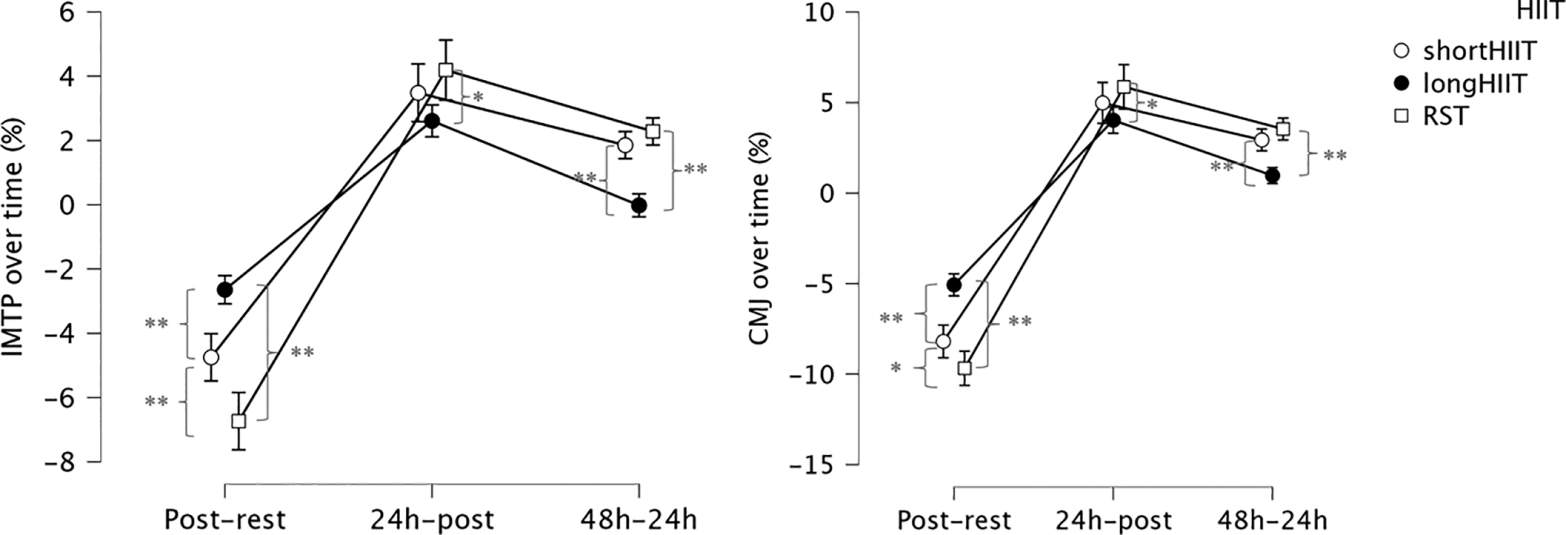
Percentages of differences between time-points in isometric mid-thigh pull (IMTP) and countermovement jump (CMJ). * Indicates significant differences between groups at the corresponding time point (p < 0.05); ** Indicates significant differences between groups at the corresponding time point (p < 0.001).

Considering the within-modality analysis, short-HIIT showed a significant decrease in IMTP from rest to post-exercise (mean difference = 1.34, p < 0.001). Recovery was observed by 24 and 48 hours post-exercise, although IMTP at 24 hours remained significantly lower than at rest (mean difference = 0.42, p = 0.006). In long-HIIT, a similar significant decrease was observed from rest to post-exercise (mean difference = 1.34, p < 0.001). IMTP at 24 hours post-exercise was significantly higher than immediately post-exercise (mean difference = -0.71, p < 0.001). Finally, in RST, significant decreases in IMTP were observed from rest to post-exercise (mean difference = 1.90, p < 0.001). Recovery was evident at 24 hours (mean difference = -1.09, p < 0.001) and further improved at 48 hours (mean difference = -1.72, p < 0.001). Variations in IMTP over time are illustrated in Figure 3.

Considering the percentage of difference between time points, time and HIIT modality was significant, F_(2.798, 268.656)_ = 29.606, p < .001, η^2^_p_ = .381, indicating that the change in IMTP percentage of difference over time differed depending on the HIIT modality. Regarding comparisons between HIIT modalities at each time point, at post exercise-rest, short-HIIT was significantly lower than long-HIIT (p < 0.001) but significantly higher than RST (p < 0.001). Additionally, long-HIIT was significantly higher than RST (p < 0.001). At post24 h-postexercise, there was no significant difference between short-HIIT and long-HIIT (p = 0.207) or between short-HIIT and RST (p = 0.411), but long-HIIT was significantly lower than RST (p = 0.004). At post48 h-post24 h, shortHIIT was significantly higher than long-HIIT (p < 0.001), there was no significant difference between short-HIIT and RST (p = 0.261), and long-HIIT was significantly lower than RST (p < 0.001).

### Countermovement jump

There was a significant main effect of time point on CMJ height, *F*_(1.823, 58.332)_ = 323.20, *p* < 0.001, η^2^_p_ = 0.957. A significant main effect of HIIT modality was also observed, *F*_(2, 64)_ = 11.64, *p* < 0.001, η^2^_p_ = 0.267. Additionally, a significant interaction effect between HIIT modality and time point was found, *F*_(2.753, 88.096)_ = 27.90, *p* < 0.001, η^2^_p_ = 0.466.

At the post-exercise time point, short-HIIT resulted in significantly greater CMJ height compared to both long-HIIT (mean difference = 0.99, *p* < 0.001) and RST (mean difference = 0.66, *p* < 0.001). Long-HIIT also showed significantly greater CMJ height than RST (mean difference = 1.65, *p* < 0.001). At 24 hours post-exercise, short-HIIT remained significantly greater than long-HIIT (mean difference = 0.69, *p* < 0.001), and long-HIIT was still significantly greater than RST (mean difference = 1.06, *p* < 0.001). No significant differences between modalities were found at 48 hours postexercise (*p* = 0.535). Variations in CMJ height are illustrated in Figure 2b.

In the within-group analysis, short-HIIT showed a significant decrease in CMJ height from rest to post-exercise (mean difference = 3.36, *p* < 0.001). Post-exercise CMJ was also significantly lower than both post-24 h (mean difference = -1.85, *p* < 0.001) and post-48 h (mean difference = -3.02, *p* < 0.001), with CMJ at 24 h significantly lower than at 48 h (mean difference = -1.17, *p* < 0.001). In long-HIIT, a significant reduction from rest to postexercise was observed (mean difference = 2.06, *p* < 0.001), and post-exercise CMJ remained significantly lower than at 24 h (mean difference = -1.54, *p* < 0.001) and 48 h (mean difference = -1.93, *p* < 0.001). Similarly, in RST, CMJ height significantly decreased from rest to post-exercise (mean difference = 3.95, *p* < 0.001). Recovery was evident by 24 h (mean difference = -1.82, *p* < 0.001) and further improved by 48 h (mean difference = -3.51, *p* < 0.001), indicating CMJ height at 48 h was significantly higher than immediately post-exercise. Furthermore, CMJ at 24 h remained significantly lower than at 48 h (mean difference = -1.38, *p* < 0.001). In practical terms, CMJ performance declined by approximately -8% after short-HIIT, -5% after long-HIIT, and -10% after RST, relative to baseline. While performance recovered close to baseline by 24 h in short- and long-HIIT, a residual suppression of -4% persisted at 24 h after RST, with only partial normalization by 48 h.

The Time and HIIT modality interaction effect was also significant, F_(2.802, 269.000)_ = 24.235, p < 0.001, η^2^_p_ = 0.336, indicating that the change in CMJ percentage of difference over time differed depending on the HIIT modality. Regarding comparisons between HIIT modalities at each time point, at postexercise-rest, short-HIIT was significantly lower than long-HIIT (p < 0.001) but significantly higher than RST (p = 0.004). Additionally, long-HIIT was significantly higher than RST (p < 0.001). At post24 h-postexercise, there was no significant difference between short-HIIT and long-HIIT (p = 0.385) or between short-HIIT and RST (p = 0.478), but long-HIIT was significantly lower than RST (p = 0.012). At post48 h-post24 h, short-HIIT was significantly higher than long-HIIT (p < 0.001), there was no significant difference between short-HIIT and RST (p = 0.178), and long-HIIT was significantly lower than RST (p < 0.001). Variations in CMJ height over time are illustrated in Figure 3b.

### Salivary interleukin-6

There was a significant main effect of time point on IL-6 levels, *F*_(1.014, 32.460)_ = 11,192.19, *p* < 0.001, η^2^_p_ = 0.997. A significant main effect of HIIT modality was also observed, *F*_(1.191, 38.106)_ = 16,156.76, *p* < 0.001, η^2^_p_ = 0.998. Additionally, a significant interaction effect between HIIT modality and time point was found, *F*_(1.981, 63.393)_ = 7,391.20, *p* < 0.001, η^2^_p_ = 0.996.

**FIG. 4:**
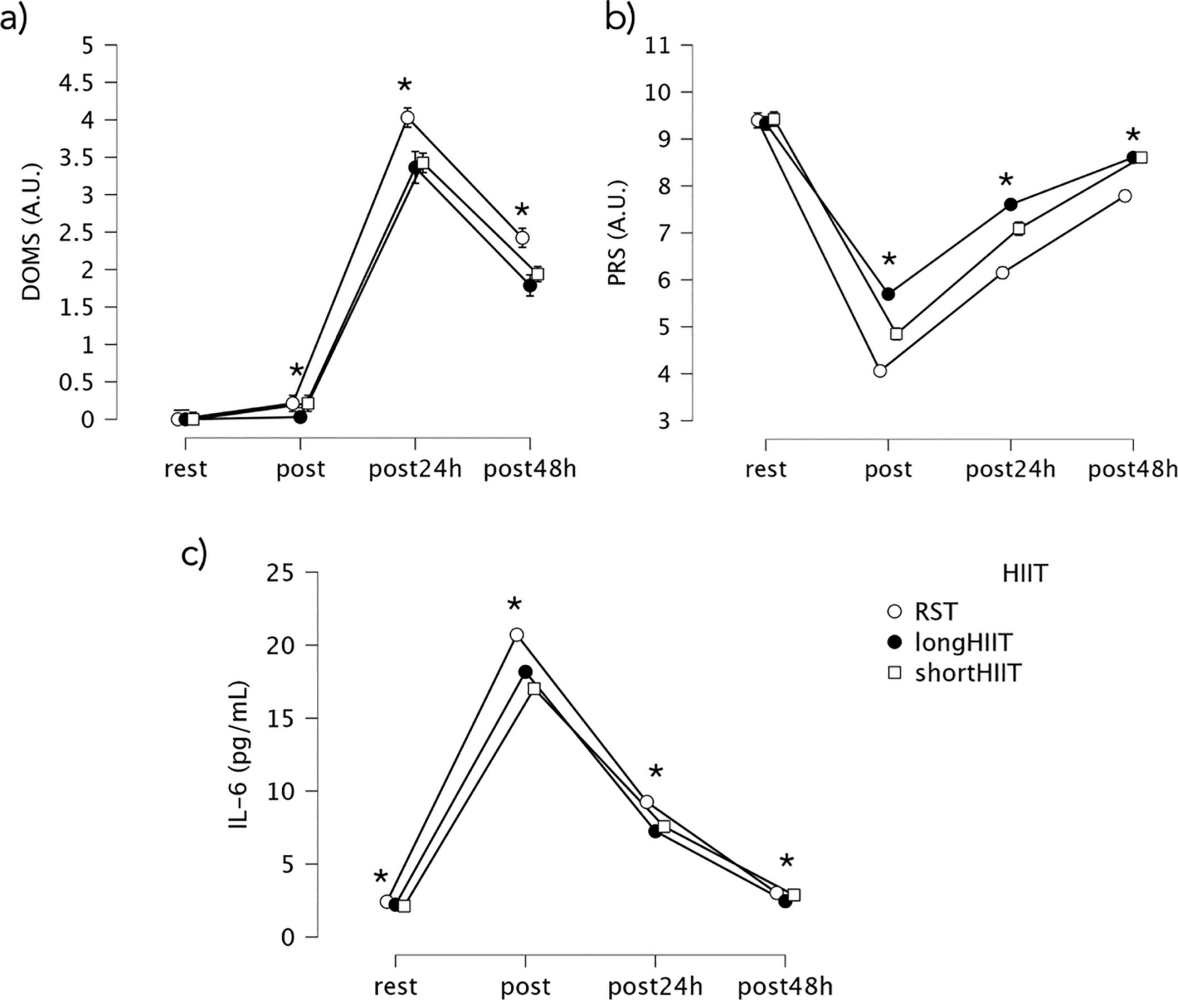
Mean values and 95% confidence intervals for variations in delayed onset muscle soreness (DOMS), Perceived recovery status scale (PRS) and salivary interleukin-6 (IL-6) across the time points. * Indicates significant differences between groups at the corresponding time point (p < 0.05).

**FIG. 5:**
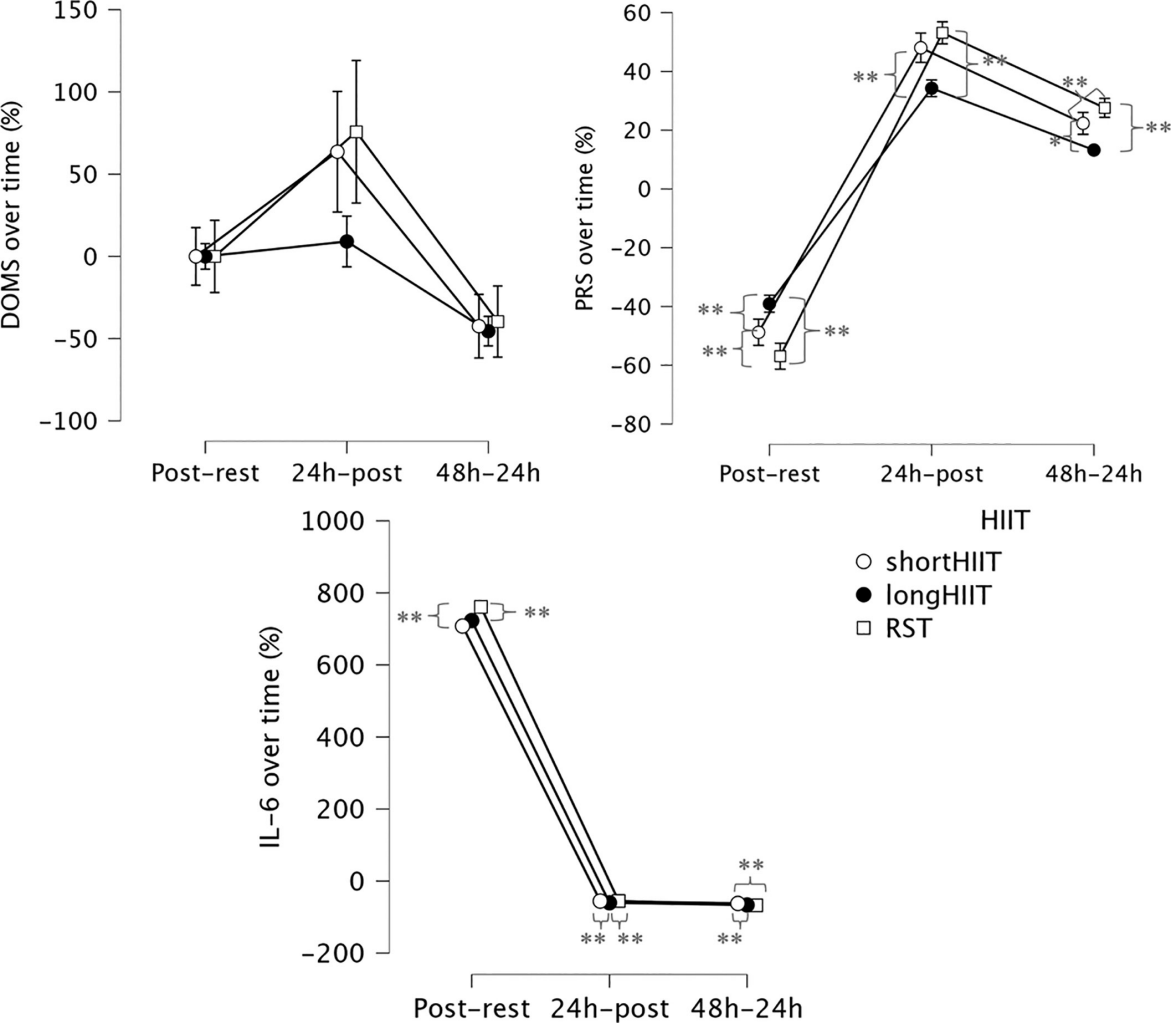
Percentages of differences between time-points in delayed onset muscle soreness (DOMS), Perceived recovery status scale (PRS) and salivary interleukin-6 (IL-6) across the time points. * Indicates significant differences between groups at the corresponding time point (p < 0.05); ** Indicates significant differences between groups at the corresponding time point (p < 0.001).

At rest, IL-6 levels in the short-HIIT group were significantly lower than those in the long-HIIT group (mean difference = -0.10, *p* < 0.001) and in the RST group (mean difference = -0.30, *p* < 0.001). Long-HIIT also showed significantly lower IL-6 levels compared to RST (mean difference = -0.20, *p* < 0.001). Immediately post-exercise, IL-6 levels remained significantly lower in short-HIIT compared to long-HIIT (mean difference = -1.16, *p* < 0.001) and RST (mean difference = -3.69, *p* < 0.001). Long HIIT also had significantly lower IL-6 levels than RST (mean difference = -2.54, *p* < 0.001). At 24 hours post-exercise, short-HIIT showed significantly higher IL-6 levels than long-HIIT (mean difference = 0.32, *p* < 0.001), indicating elevated IL-6 in short-HIIT at this time point. However, IL-6 levels in short-HIIT were still significantly lower than those in RST (mean difference = -1.69, *p* < 0.001), and long-HIIT also remained significantly lower than RST (mean difference = -2.01, *p* < 0.001). At 48 hours post-exercise, short-HIIT continued to show significantly higher IL-6 levels compared to long-HIIT (mean difference = 0.42, *p* < 0.001), but levels remained significantly lower than RST (mean difference = -0.15, *p* < 0.001). Long-HIIT was also significantly lower than RST (mean difference = -0.57, *p* < 0.001). Variations in IL-6 are illustrated in Figure 4c and over time Figure 5c.

Within-group comparisons revealed that in the short-HIIT group, IL-6 levels significantly increased from rest to post-exercise (mean difference = -14.90, *p* < 0.001), and then significantly decreased at 24 h (mean difference = 9.45, *p* < 0.001) and 48 h (mean difference = 14.15, *p* < 0.001) post-exercise. IL-6 levels at 24 h were also significantly greater than at 48 h (mean difference = 7.55, *p* < 0.001). In the long-HIIT group, IL-6 also significantly increased from rest to post-exercise (mean difference = -15.96, *p* < 0.001). Post-exercise levels were significantly higher than at both 24 h (mean difference = 10.93, *p* < 0.001) and 48 h (mean difference = 15.72, *p* < 0.001), with IL-6 at 24 h still significantly higher than at 48 h (mean difference = 5.03, *p* < 0.001). In the RST group, IL-6 levels significantly increased from rest to post-exercise (mean difference = -18.29, *p* < 0.001). Post-exercise IL-6 was significantly higher than at 24 h (mean difference = 11.46, *p* < 0.001) and 48 h (mean difference = 17.69, *p* < 0.001), with a significant decrease also observed from 24 h to 48 h (mean difference = 6.84, *p* < 0.001).

The interaction between time and HIIT modality was significant, F_(2.001, 192.096)_ = 12.209, p < 0.001, ηp2 = 0.203, indicating that the change in IL-6 percentage of difference over time differed depending on the HIIT modality. Regarding comparisons between HIIT modalities at each time point, at postexercise-rest, there was no significant difference between short-HIIT and long-HIIT (p = 0.510), but short-HIIT was significantly lower than RST (p < 0.001). Additionally, long-HIIT was significantly lower than RST (p = 0.003). At post24 h-postexercise, short-HIIT was significantly higher than long-HIIT (p < 0.001), there was no significant difference between short-HIIT and RST (p = 1.000), but long-HIIT was significantly lower than RST (p < 0.001). At post48 h-post24 h, short-HIIT was significantly higher than long-HIIT (p < 0.001) and significantly higher than RST (p < 0.001). There was no significant difference between long-HIIT and RST (p = 0.081).

### Delayed onset muscle soreness

The Friedman test revealed a statistically significant effect of modality and time point on DOMS levels, χ^2^(11) = 347.455, *p* < 0.001. At rest, no significant differences were observed between any of the HIIT modalities (Z = 0.000, *p* = 1.000 for all comparisons), as DOMS scores were zero across all groups. However, immediately post-exercise, long-HIIT showed significantly lower DOMS compared to short-HIIT (Z = -2.121, *p* = 0.034) and RST (Z = -2.121, *p* = 0.034). At 24 hours post-exercise, RST presented significantly higher DOMS compared to both short-HIIT (Z = -4.472, *p* < 0.001) and long-HIIT (Z = -3.581, *p* < 0.001). At 48 hours post-exercise, RST continued to show significantly higher DOMS compared to short-HIIT (Z = -4.000, *p* < 0.001) and long-HIIT (Z = -3.666, *p* < 0.001). Variations in DOMS over time are illustrated in Figure 4a. At rest, DOMS was 0 (median = 0, IQR = 0–0) across all modalities. Immediately post-exercise, DOMS remained minimal (median < 0.3). At 24 h, DOMS peaked, with RST showing the highest values (median = 4.0, IQR = 3.7–4.4) compared with short-HIIT (median = 3.4, IQR = 3.1–3.8) and long-HIIT (median = 3.4, IQR = 2.8–4.0). By 48 h, DOMS declined but remained greater in RST (median = 2.4, IQR = 2.1–2.7) relative to short- and long-HIIT (medians ≈1.8–1.9). These values suggest that, although statistically significant, the practical severity of DOMS was modest, reflecting mild-tomoderate soreness overall.

Within-group comparisons revealed that in the short-HIIT condition, there was a significant increase in DOMS from rest to post-exercise (Z = -2.646, *p* = 0.008). DOMS continued to rise significantly from post-exercise to 24 hours (Z = -5.340, *p* < 0.001), reaching its peak at 24 h, before significantly decreasing by 48 h (Z = -5.169, *p* < 0.001). All post-rest time points (post-exercise, 24 h, and 48 h) showed significantly greater DOMS compared to rest (*p* < 0.001 for all).

In the long-HIIT condition, no significant difference was observed between rest and post-exercise (Z = -1.000, *p* = 0.317). However, DOMS significantly increased from rest to 24 h (Z = -5.126, *p* < 0.001), and from post-exercise to 24 h (Z = -5.120, *p* < 0.001). DOMS at 24 h was significantly higher than at 48 h (Z = -5.064, *p* < 0.001), and DOMS at 48 h remained significantly greater than at both rest and post-exercise (*p* < 0.001 for both comparisons).

In the RST condition, DOMS significantly increased from rest to post-exercise (Z = -2.646, *p* = 0.008), and continued to rise from post-exercise to 24 h (Z = -5.301, *p* < 0.001). DOMS peaked at 24 h before significantly decreasing at 48 h (Z = -5.190, *p* < 0.001). All post-rest time points (post-exercise, 24 h, and 48 h) were significantly greater than rest (*p* < 0.001 for all comparisons).

### Perceived recovery status scale

The Friedman test revealed a statistically significant effect of exercise modality and time point on PRS levels, χ^2^(11) = 354.544, p < 0.001. Between-group comparisons at each time point showed no significant differences between modalities at rest (p > 0.05). However, immediately post-exercise, long-HIIT resulted in significantly greater PRS compared to both short-HIIT (Z = -5.292, p < 0.001) and RST (Z = -5.205, p < 0.001). Short-HIIT also showed significantly greater PRS than RST (Z = -5.099, p < 0.001). At 24 hours post-exercise, long-HIIT maintained significantly higher PRS than both short-HIIT (Z = -4.123, p < 0.001) and RST (Z = -5.172, p < 0.001), while short-HIIT also showed significantly higher PRS than RST (Z = -5.568, p < 0.001). At 48 hours post-exercise, no significant difference was found between short HIIT and long-HIIT (Z = 0.000, p = 1.000). However, both modalities showed significantly greater PRS compared to RST (short-HIIT vs. RST: Z = -5.196, p < 0.001; long-HIIT vs. RST: Z = -5.196, p < 0.001), indicating better perceived recovery in both HIIT conditions relative to RST. Variations in PRS over time are illustrated in Figure 4b.

Within-group comparisons showed that in the short-HIIT condition, PRS significantly decreased from rest to post-exercise (Z = -5.192, p < 0.001), followed by a significant increase from postexercise to 24 h (Z = -5.304, p < 0.001), and another significant increase from 24 h to 48 h (Z = -5.304, p < 0.001), suggesting progressive recovery. However, all post-rest time points remained significantly lower than rest (p < 0.001), indicating perceived recovery did not return to baseline within 48 h.

In the long-HIIT condition, PRS also significantly declined from rest to post-exercise (Z = -5.218, p < 0.001), then significantly increased from post-exercise to 24 h (Z = -5.533, p < 0.001), and again from 24 h to 48 h (Z = -5.745, p < 0.001). As with short-HIIT, PRS at all post-rest time points remained significantly lower than at rest (p < 0.001), indicating incomplete recovery.

In the RST condition, PRS significantly decreased from rest to post-exercise (Z = -5.151, p < 0.001), followed by significant increases from post-exercise to 24 h (Z = -5.533, p < 0.001), and from 24 h to 48 h (Z = -5.205, p < 0.001). Yet, PRS at all postrest time points was still significantly lower than at rest (p < 0.001), indicating a similar delayed recovery pattern.

## DISCUSSION

The present study aimed to evaluate the acute and sub-acute neuromuscular fatigue and inflammatory responses in middle-distance runners following short-interval HIIT, long-interval HIIT, and RST. Our findings showed that while all HIIT modalities induced transient decrements in performance, increased inflammation, and altered perceived recovery, RST promoted a more marked and prolonged physiological and perceptual recovery demand compared to short- and long-HIIT. These results highlight the differential impact of various HIIT modalities on middle-distance runners. A novelty of this study is the direct comparison of three HIIT modalities—short-interval, long-interval, and RST—within the same group of middle-distance runners using a crossover design. While previous studies [19, 34] have often examined a single protocol or focused on acute responses, our findings extend the literature by showing that RST induces more prolonged neuromuscular fatigue, inflammation, and perceived soreness compared to both short- and long-interval HIIT. Furthermore, the combined use of objective (CMJ, IMTP), physiological (IL-6), and subjective (DOMS, PRS) measures provides a multidimensional assessment of residual fatigue, offering a more diverse characterization than prior HIIT research.

Regarding the IMTP performance, our study showed that immediately post-exercise, all three groups experienced significant reductions in IMTP force from baseline, with RST showing the greatest decrement. Long-HIIT also showed significantly lower IMTP compared to short-HIIT post-exercise, and RST was significantly lower than both short- and long-HIIT. This acute effect was further evidenced by the percentage change from rest to post-exercise, where RST exhibited a proportionally larger reduction compared to short-HIIT, and long-HIIT also showed a greater decline than short-HIIT. These findings align with previous research that reported approximately a 10% reduction in maximal voluntary contraction following RST [35]. Additionally, another study observed impairments in neuromuscular function after HIIT, although recovery was noted within 24 hours, including normalization of running kinematics and neuromuscular performance [11]. At 24 hours post-exercise, short-HIIT showed better IMTP recovery compared to long-HIIT and RST, with only minor residual IMTP values from baseline, while RST still exhibited significant reductions. The analysis of percentage recovery from post-exercise to 24 hours also indicated a slower recovery rate for RST compared to long-HIIT. By 48 hours, IMTP values for short- and long-HIIT had returned to baseline, but RST still showed a statistically significant, albeit smaller, decline compared to short-HIIT. This prolonged decline of maximal force production after RST may be associated with greater strain imposed by repeated maximal running, likely involving higher eccentric loading, and with a greater contribution of central fatigue that could limit voluntary activation [36]. However, we did not directly assess markers of muscle damage (e.g., creatine kinase, muscle ultrasound) or central fatigue (e.g., EMG, voluntary activation techniques). Therefore, these explanations remain speculative, and future studies incorporating such measures are warranted. The higher mechanical stress and potential for muscle damage inherent in sprinting may lead to more substantial peripheral fatigue. However, the absence of changes in direction (i.e., no turns) likely mitigated the extent of impairment, as suggested by findings from a previous study [37].

The CMH data followed the IMTP findings, showing that all three HIIT modalities significantly reduced jump height immediately postexercise. RST led to the most substantial acute CMJ decrement, followed by long-HIIT, and then short-HIIT, which aligns with the common observation of impaired vertical jump performance after demanding training sessions due to acute fatigue. The percentage change from rest to post-exercise confirmed RST’s significantly greater acute decline compared to both short- and long-HIIT. This immediate reduction in CMJ is a regularly reported indicator of lower limb power output impairment following various forms of high-intensity exercise [13], namely in those more intense as sprint, causing an impairment on force-time curves [38]. The recovery trajectory of CMJ height revealed that, at 24 hours post-exercise, values remained significantly lower for the RST condition compared to both short- and long-interval HIIT, indicating a slower recovery of explosive power. The slower recovery rate for RST from post-exercise to 24 hours was also reflected in the percentage difference analysis compared to long-HIIT. Although all modalities showed improvement by 48 hours, CMJ performance in the RST group remained lower, albeit not statistically different from the other conditions at this time point. From a practical perspective, the magnitude of the residual CMJ decrement after RST (~2–3 cm or 5–7% lower than baseline at 24 h) may still represent a meaningful reduction in explosive performance for middle-distance runners, potentially impairing training quality if another high-intensity session is scheduled too soon. In contrast, the smaller decrements observed after short- and long-HIIT (≤ 1–2 cm, ~2–4%) are less likely to meaningfully affect subsequent training. This prolonged recovery pattern follows the trends observed in IMTP performance and is consistent with previous studies reporting extended neuromuscular impairment following more demanding and about to close maximal speeds [39]—likely due to the combined neuromuscular and metabolic demands of such efforts. For example, a repeated sprint protocol can cause significant muscle damage, with elevated creatine kinase and muscle soreness lasting up to 72 hours post-exercise [40]. This impairment is not solely attributable to central fatigue; it may also reflect an impact on the stretchshortening cycle by reducing reflex contribution [41] due to the possible mechanical load imposed by the all-out efforts.

The analysis of IL-6 levels revealed that all three protocols led to significant post-exercise increases from baseline, with RST showing the highest spike—significantly exceeding both long- and short-interval HIIT. The percentage increase from rest to post-exercise corroborated this, showing RST induced a proportionally larger acute inflammatory response. This elevation may be linked to increased myokine release [42] triggered by the high mechanical stress of allout efforts, with IL-6 acting as a signaling molecule to mobilize energy substrates [43]. The greater magnitude of IL-6 release in RST could also be attributed to the larger amount of muscle mass recruited [44] during sprinting. At 24 hours post-exercise, IL-6 levels remained significantly elevated across all conditions, with RST continuing to show the highest values, suggesting a more prolonged inflammatory response compared to the other two modalities. While the percentage recovery from post-exercise to 24 hours was similar for short-HIIT and RST, the recovery rate from 24 hours to 48 hours in RST was significantly slower than short-HIIT, suggesting a more prolonged inflammatory response compared to the other two modalities. Although levels generally declined by 48 hours, RST still presented with residual elevation. It should be noted, however, that our study only followed athletes for 48 hours; whether IL-6 levels fully normalize or continue to diverge beyond this period remains unknown, and any extrapolation past 48 h should be made with caution. While our study did not include specific physiological markers to directly confirm the possible mechanisms, one possible explanation for the IL-6 response is the higher metabolic stress induced by RST, taxing greater eccentric damage [45]. Additionally, the greater reliance on anaerobic glycolysis during RST [46] may lead to increased lactate accumulation and a more acidic intramuscular environment [47], which could further stimulate IL-6 production. However, it should also be acknowledged that nutritional factors (e.g., pre-exercise glycogen availability, carbohydrate intake during recovery) can strongly modulate IL-6 responses [48, 49], and diet was not strictly standardized in our study. This lack of dietary control may have contributed to variability in IL-6 levels and represents an important limitation when interpreting these findings. These interpretations therefore remain speculative given the limitations of our current study design and measured outcomes.

Immediately post-exercise, DOMS scores were minimal across all modalities. However, long-HIIT showed slightly lower DOMS immediately post-exercise compared to short-HIIT and RST. The most significant differences emerged at 24 hours post-exercise, where RST elicited significantly higher DOMS compared to both short- and long-HIIT. This elevated DOMS persisted at 48 hours in RST, indicating a prolonged subjective experience of muscle damage. The PRS showed significantly lower values immediately post-exercise and at 24 hours, indicating a poorer subjective state of readiness. While PRS improved across all modalities by 48 hours, both short- and long-HIIT revealed significantly higher PRS than RST. Considering impairments in IMTP, CMJ and IL-6, the slower recovery for RST, can be attributed to the extent of exercise-induced muscle damage likely promoted by the repeated sprints and strain, which aligns with a previous review [50]. The possibly greater eccentric contractions and high mechanical forces in RST were possibly drivers for muscle micro-trauma leading to DOMS, as previously observed [40]. This physical discomfort, promoted by the increased DOMS, possibly influenced the athlete’s perceived recovery. The elevation of IL-6 observed in RST may also support the notion of more extensive tissue perturbation, contributing to both the subjective experience of soreness and the objective indicators of recovery as observed in a previous study conducted in plyometric training [51].

This study is not without limitations. First, the participants were exclusively male middle-distance runners, which improved sample homogeneity but limits the generalizability of the findings, as biological sex may influence neuromuscular and inflammatory responses [52]. Future research should therefore include female athletes to examine potential sex-specific adaptations. Second, although salivary IL-6 served as a non-invasive indicator of inflammation, incorporating additional biomarkers (e.g., C-reactive protein, creatine kinase, or muscle biopsy data) could provide a more comprehensive understanding of exercise-induced stress and recovery. Third, recovery was only monitored up to 48 hours post-exercise; extending this window to 72 hours or longer would help capture potential lingering effects of different HIIT modalities. Fourth, dietary intake, sleep patterns, and other recovery-related behaviors were not strictly controlled, although participants were instructed to refrain from training for 48 hours before and after each HIIT session, and all sessions were scheduled at the same time of day to minimize circadian influences. Fifth, although each HIIT modality was separated by at least 7 days, participants continued their regular training during this period. As such, residual effects or training-induced variability cannot be entirely ruled out. Sixth, intensities were prescribed from a single baseline ${\text{v}\dot{\text{V}}\text{O}}_{2\max}$ assessment. Although this approach avoided repeated testing and potential fatigue, fluctuations in aerobic capacity across the study period may have introduced minor variability in workload between sessions. Finally, we note that a standardized warm-up was included before the baseline, 24 h, and 48 h assessments, but not immediately post-HIIT, since athletes were already physiologically warmed up. Although this may introduce slight differences in testing context, the approach reflects real-world conditions and minimizes unnecessary fatigue after the training sessions.

Despite these limitations, this study provides novel insight into recovery kinetics across three HIIT modalities in middle-distance runners. RST appears to induce a more prolonged state of neuromuscular fatigue, inflammation, and perceived soreness compared to both short- and long-HIIT. Coaches should anticipate that athletes may require extended recovery periods—potentially exceeding 48 hours—for complete neuromuscular and subjective recovery following RST sessions. While responses beyond 48 h were not measured, it may be prudent to avoid scheduling another high-intensity session or competition within 48–72 h of RST. Accordingly, RST may be better suited to preparatory phases, when developing anaerobic and neuromuscular qualities is prioritized, rather than during competition periods where rapid recovery is essential. This information can inform training periodization, helping to schedule lower-intensity days between high-intensity sessions. Additionally, coaches can monitor both subjective feedback—such as daily DOMS and PRS scores—and objective performance indicators like CMJ or IMTP values to better gauge recovery status and determine whether extended recovery is necessary.

## CONCLUSIONS

This study suggests that HIIT elicits modality-specific acute and sub-acute responses in middle-distance runners. Specifically, RST induced greater acute decrements in neuromuscular performance (IMTP and CMJ), higher inflammatory responses (IL-6), and a more pronounced perception of fatigue and soreness (lower PRS, higher DOMS) compared to short- and long-HIIT. The recovery following RST was longer, with significant physiological and subjective markers of fatigue persisting up to 48 hours post-exercise. While our findings indicate that RST imposes a more substantial and prolonged physiological impact than other HIIT formats within this timeframe, it remains uncertain whether full recovery occurs by 48 h or requires longer. Therefore, caution is advised when scheduling subsequent high-intensity sessions after RST, but recommendations beyond 48 h cannot be confirmed from the present data.
